# Tigray Orthohantavirus Infects Two Related Rodent Species Adapted to Different Elevations in Ethiopia

**DOI:** 10.1089/vbz.2019.2452

**Published:** 2019-11-27

**Authors:** Yonas Meheretu, William T. Stanley, Evan W. Craig, Joëlle Goüy de Bellocq, Josef Bryja, Herwig Leirs, Meike Pahlmann, Stephan Günther

**Affiliations:** ^1^ Department of Biology and Institute of Mountain Research and Development, Mekelle University, Mekelle Ethiopia.; ^2^ Field Museum of Natural History, Chicago, Illinois.; ^3^ Environmental Studies, Antioch University New England, Keene, New Hampshire.; ^4^ Department of Population Biology, Institute of Vertebrate Biology of the Czech Academy of Sciences, Brno, Czech Republic.; ^5^ Evolutionary Ecology Group, University of Antwerp, Antwerp, Belgium.; ^6^ Department of Virology, Bernhard Nocht Institute for Tropical Medicine, Hamburg, Germany.

**Keywords:** orthohantavirus, *Stenocephalemys*, Afroalpine, phylogeny, Simien Mountains

## Abstract

Orthohantaviruses are RNA viruses that some members are known to cause severe zoonotic diseases in humans. Orthohantaviruses are hosted by rodents, soricomorphs (shrews and moles), and bats. Only two orthohantaviruses associated with murid rodents are known in Africa, Sangassou orthohantavirus (SANGV) in two species of African wood mice (*Hylomyscus*), and Tigray orthohantavirus (TIGV) in the Ethiopian white-footed rat (*Stenocephalemys albipes*). In this article, we report evidence that, like SANGV, two strains of TIGV occur in two genetically related rodent species, *S. albipes* and *S*. sp. A, occupying different elevational zones in the same mountain. Investigating the other members of the genus *Stenocephalemys* for TIGV could reveal the real diversity of TIGV in the genus.

## Introduction

African orthohantaviruses have received very little attention until recently (Klempa et al. 2006, Kang et al. 2014, Těšíková et al. 2017). In 2012, Ethiopian white-footed rats (*Stenocephalemys albipes*) trapped from peridomestic areas in Ethiopia were found to carry a novel orthohantavirus called Tigray orthohantavirus (TIGV; Meheretu et al. 2012). Later on, the complete genome of TIGV has been characterized, revealing a typical orthohantavirus organization (Goüy de Bellocq et al. 2016).

Cases of genetically related orthohantaviruses occurring in genetically related mammalian host species have been well reported (Milholland et al. 2018). However, such cases are limited to African orthohantaviruses in rodents and shrews. The description of the sister lineage to the Sangassou orthohantavirus from *Hylomyscus endorobae* in Kenya in a rodent host phylogenetically related to the *Hylomyscus simus* in Guinea represents a case of virus lineage divergence separated by large geographical distance (Tesikova et al. 2017). Kang et al. (2014) described two new hantaviruses from the Geata mouse shrew (*Myosorex geata*) and Kilimanjaro mouse shrew (*Myosorex zinki*) captured in Tanzania. In this study, we provide additional evidence for such pattern in an African orthohantavirus. Two strains of TIGV are reported to occur in two genetically related rodent species occupying different elevational zones in the highest mountains in Ethiopia.

## Materials and Methods

We obtained 94 dry blood spots on calibrated prepunched filter paper (LDA 22, Ploufragan, France) from two species of *Stenocephalemys* rats sampled from four elevations within Simien Mountains National Park (SMNP) in Ethiopia, between September and November 2015 during a small mammal biodiversity survey by the Field Museum of Natural History, Chicago (USA) and Mekelle University (Ethiopia). The trapping elevations were 2900 meters above sea level (13.19°N, 37.89°E): 3250 meters (13.23°N, 38.04°E), 3600 meters (13.26°N, 38.19°E), and 4000 meters (13.17°N, 38.20°E).

Total viral RNA was extracted from the dry blood spots using QIAamp viral RNA Mini Kit (Qiagen). The RNA samples were screened for the orthohantavirus RNA by an optimized assay (Supplementary Data S1) targeting partial polymerase (L) gene sequences of the orthohantavirus genomes using primers Han-L-F1: 5′-ATGTAYGTBAGTGCWGATGC-3′; Han-L-R1: 5′-AACCADTCWGTYCCRTCATC-3′; Han-L-F2: 5′-TGCWGATGCHACIAARTGGTC-3′; and Han-L-R2: 5′-GCRTCRTCWGARTGRTGDGCAA-3′ (Klempa et al. 2006). The PCR products were Sanger sequenced with both forward and reverse primers. Sequences were submitted to GenBank under accession numbers MK875668, MK875669, MK875670, MK875672, MK875673, and MK875674.

Sequences were edited in Geneious 8.0.5 and aligned with the full coding part of L segment sequences of representative orthohantaviruses. Phylogenetic analyses were performed by the maximum likelihood (ML) approach in PhyML 3.1 (Guindon et al. 2010) and Bayesian inference was implemented in MrBayes 3.2.2 (Ronquist et al. 2012) using GTR+I+Γ substitution model. For the ML tree, support was evaluated by 1000 replicate bootstraps. In MrBayes, we used the default priors for all parameters and two independent runs were conducted with 1,000,000 generations per run; trees and parameters were sampled every 500 generations. Bayesian posterior probabilities were used to assess branch support. Thottapalayam and Uluguru viruses were used as out-groups. Trees were visualized and annotated in FigTree, version 1.4.1. (http://tree.bio.ed.acuk/software/figtree/). Genetic *p* distances were estimated in MEGA7 (Kumar et al. 2016). Host identification was performed by genotyping of nuclear and mitochondrial markers as described in Bryja et al. (2018).

## Results and Discussion

Of the 94 investigated hosts, 6 individuals (6.38%) were positive for TIGV. The total TIGV prevalence in *S. albipes* was 11.11% (3/27): 12.5% (1/8) at 2900 meters and 10.53% (2/19) at 3250 meters elevations where this species only occurred. This prevalence was lower than reported previously for *S. albipes* at lower elevations in peridomestic areas (17.85%, *i.e.*, 10/56; Meheretu et al. 2012). Total TIGV prevalence in *S.* sp. A was 4.48% (3/67): 10.53% (2/19) at 3600 meters and 2.08% (1/48) at 4000 meters elevations where this species only occurred.

Interestingly, positive individuals were found at all four sampling elevations. Bryja et al. (2018) showed the existence of six genetically supported species under the genus *Stenocephalemys*. Geographically, *S. albipes* is the most widespread species in Ethiopia, whereas *S.* sp. A is reported only from the highest elevations in four isolated mountain tops of northwestern Ethiopian highlands. In the SMNP, only two species, likely evolved by ecological speciation (Bryja et al. 2018), were genetically identified: *S. albipes* occurring from Montane forest to the ecotone extending to the *Erica* shrub at the lower elevations and *S.* sp. A in the belt of *Erica* shrub and the Afroalpine meadow at higher elevation.

**FIG. 1.**
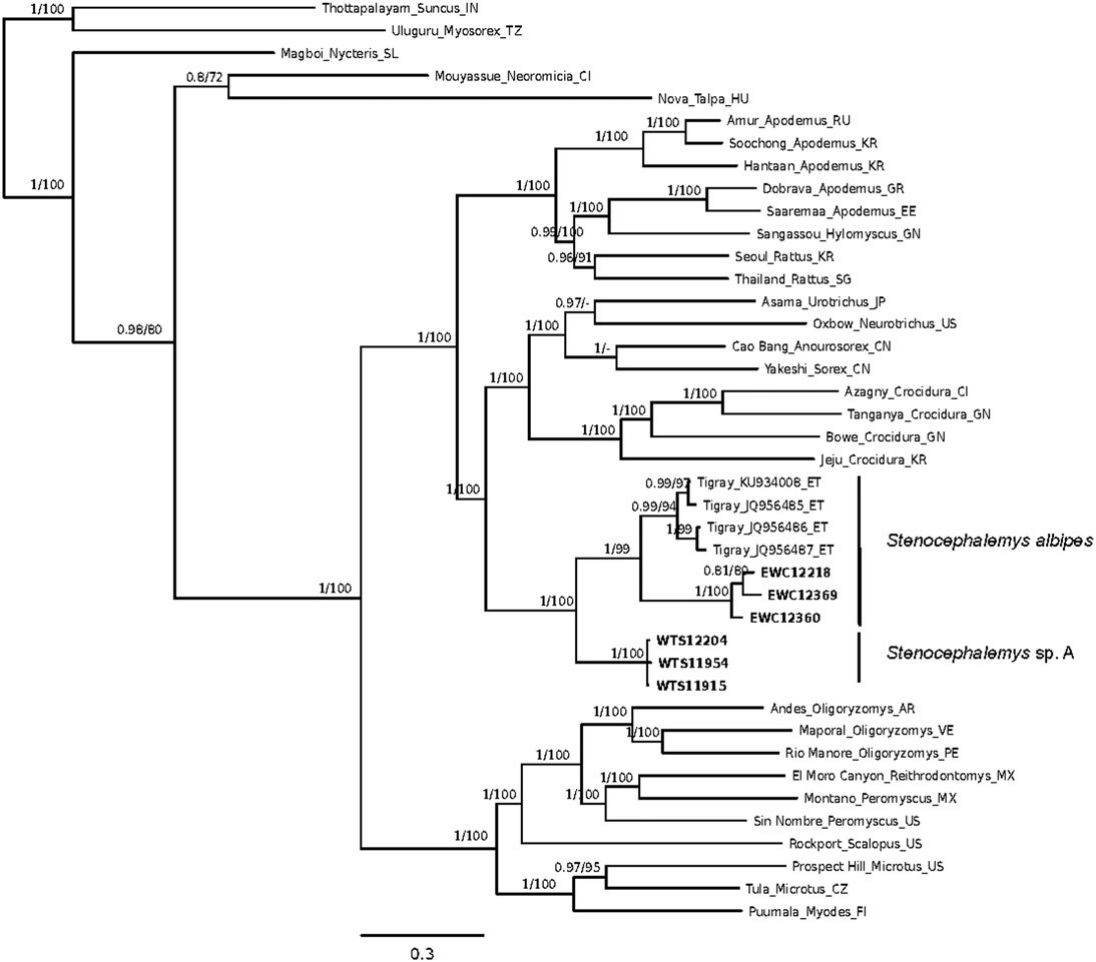
Phylogenetic tree estimated from the Bayesian analysis of the complete (when available) L coding part of representative orthohantaviruses and the new Tigray sequences (347 nucleotides long) using the GTR+I+Γ model of evolution. Since tree topologies were very similar between PhyML and MrBayes, only MrBayes tree is shown. Analyzed viruses with GenBank accession numbers are given in Supplementary Data S2. Numbers above branches represent Bayesian posterior probability/ML bootstrap support (1000 replicates). The small scale bar represents the number of nucleotide substitutions per site. Names of viruses are given in full followed by the host genus and ISO code of the country. Sequences from this study are in *bold*. ML, maximum likelihood.

The ML and Bayesian analyses using representative sequences from other orthohantaviruses, four TIGV sequences already available in GenBank and the six sequences from this study, produced trees with similar topologies (one exception being a soricomorpha-borne orthohantavirus clade—see Fig. 1). The sequences grouped by host species, the TIGV from *S.* sp. A clade being sister to the TIGV from *S. albipes* clades from Tigray and SMNP.

The number of nucleotide and amino acid differences per site from averaging overall sequence pairs between the two TIGV clades from *S. albipes* is 19.04% ± 1.94% and 4.82% ± 1.95%, respectively. The divergence between TIGV clades from *S. albipes* and *S.* sp. A is 22.32% ± 1.88% and 7.65% ± 2.34% at nucleotides and amino acids, respectively. Finally, the divergence between *S. albipes* and *S*. sp. A clades from SMNP is 23.12% ± 2.15% and 8.46% ± 2.64% nucleotides and amino acids, respectively. Therefore, these orthohantaviruses seem to be two strains of TIGV carried by two genetically related rodent species occurring on the same mountain but at different elevations.

## Conclusion

We report the occurrence of TIGV orthohantavirus in two sister host species that evolved by ecological speciation at different elevational zones in the Simien Mountains. It should be noted that both allopatric (*i.e.*, *Hylomyscus*) and ecological (*i.e.*, *Stenocephalemys*) speciation of rodents can be accompanied by codivergence of their orthohantaviruses. Investigating whether the other members of the genus *Stenocephalemys* could also carry TIGV would be an area of future interest to understand how the virus is maintained in *multiple species*. Eventually, TIGV could be used as a model candidate virus to investigate host–virus codivergence scenarios.

## Supplementary Material

Supplemental data

Supplemental data
