# Supplementary material for: Tigray Orthohantavirus Infects Two Related Rodent Species Adapted to Different Elevations in Ethiopia
Source: Vector Borne Zoonotic Dis. 2019 Nov 27;19(12):950–3. doi: 10.1089/vbz.2019.2452 (PMC6882452; doi:10.1089/vbz.2019.2452)
Supplement: Supplemental data [file Supp_Data1.pdf]

Supplementary Data

Supplementary Data S1

Optimized PCR assay

Viral RNA was extracted from dry blood spots using Qiagen viral RNA kit (Qiagen) as recommended. The RNA samples were screened for the orthohantavirus RNA by an optimized assay targeting partial polymerase (L) gene sequences of the orthohantavirus genomes using primers Han-L-F1: 5′-ATGTAYGTBAGTGCWGATGC-3′; Han-L-R1: 5′-AACCADTCWGTYCCRTCATC-3′; Han-L-F2: 5′-TGC WGATGCHACIAARTGGTC-3′; and Han-L-R2: 5′-GCRT CRTCWGARTGRTGDGCAA-3′ (Klempa et al., 2006). We optimized the amplification assay where a first-round PCR (Qiagen one-step RT-PCR kit) of 25 μL contained 5 μL of RNA, 1 × buffer, 1 × Q-solution, 0.4 mM of each deoxynucleoside triphosphate (dNTP), 0.6 mM of HAN-L-F1 and HAN-L-R1 primers, and 1 μL enzyme mix. Cycling was then done on a Primus 25 advanced cycler (PeqLab, Erlangen, Germany) at 50°C for 30 min; 95°C for 15 min, followed by 45 cycles of 95°C for 30 s, 48°C for 1 min, and 72°C for 1 min. The second-round PCR (Phusion High-Fidelity DNA Polymerase kit; New England Biolabs) of 25-μL reaction volume contained 2 μL of first-round PCR product, 1 × phusion buffer, 0.2 mM of dNTP, 0.5 mM Han-L-F2 and Han-L-R2 primers, and 0.25 μL enzyme. The second-round PCR comprised 98°C for 30 s; 35 cycles of 98°C for 10 s, 50°C for 30 s, and 72°C for 30 s; and 72°C for 7 min.

Supplementary Reference

Klempa B, Fichet-Calvet E, Lecompte E, Auste B, et al. Hantavirus in African wood mouse, Guinea. Emerg Infect Dis 2006; 12:838–840.
